# Optimizing Deep Learning Models for Luminal and Nonluminal Breast Cancer Classification Using Multidimensional ROI in DCE‐MRI—A Multicenter Study

**DOI:** 10.1002/cam4.70931

**Published:** 2025-05-10

**Authors:** Zhenfeng Huang, Zhikun Qiu, Shuyuan Chen, Yideng Zhang, Kunyi Wang, Qingan Zeng, Yukang Huang, Yusong Zhang, Juyuan Bu

**Affiliations:** ^1^ Department of Thyroid & Breast Surgery The Fifth Affiliated Hospital, Sun Yat‐sen University Zhuhai China; ^2^ Guangdong Provincial Engineering Research Center of Molecular Imaging, the Fifth Affiliated Hospital Sun Yat‐sen University Zhuhai China; ^3^ Department of Breast Surgery Huizhou Central People's Hospital Huizhou China; ^4^ Linyi People's Hospital Linyi China; ^5^ Department of Gastrointestinal Surgery, the Fifth Affiliated Hospital Sun Yat‐Sen University Zhuhai China

**Keywords:** breast cancer, DCE‐MRI, multidimensional, ROI only, ROI original

## Abstract

**Objectives:**

Previous deep learning studies have not explored the synergistic effects of ROI dimensions (2D/2.5D/3D), peritumoral expansion levels (0–8 mm), and segmentation scenarios (ROI only vs. ROI original). Our study aims to evaluate the performance of multidimensional deep transfer learning models in distinguishing molecular subtypes of breast cancer (luminal vs. nonluminal) using DCE‐MRI. Under two segmentation scenarios, we systematically compare the effects of ROI dimensions and peritumoral expansion levels to optimize multidimensional deep learning models via transfer learning for distinguishing luminal from nonluminal breast cancers in DCE‐MRI‐based analysis.

**Materials and Methods:**

From October 2020 to October 2023, data from 426 patients with primary invasive breast cancer were retrospectively collected. Patients were divided into three cohorts: (1) training cohort, *n* = 108, from SYSU Hospital (Zhuhai, China); (2) validation cohort 1, *n* = 165, from HZ Hospital (Huizhou, China); and (3) validation cohort 2, *n* = 153, from LY Hospital (Linyi, China). ROIs were delineated, and expansions of 2, 4, 6, and 8 mm beyond the lesion boundary were performed. We assessed the performance of various deep transfer learning models, considering precise segmentation (ROI only and ROI original) and varying peritumoral regions, using ROC curves and decision curve analysis.

**Results:**

The 2.5D1‐based deep learning model (ROI original, 4 mm expansion) demonstrated optimal performance, achieving an AUC of 0.808 (95% CI 0.715–0.901) in the training cohort, 0.766 (95% CI 0.682–0.850) in validation cohort 1, and 0.799 (95% CI 0.725–0.874) in validation cohort 2.

**Conclusion:**

The study highlights that the 2.5D1‐based deep learning model utilizing the three principal slices of the minimum bounding box (ROI original) with a 4 mm peritumoral region is effective in distinguishing between luminal and nonluminal breast cancer tumors, serving as a potential diagnostic tool.

**Abbreviations**
AUC: the area under the curve
DCA: decision curve analysis
DCE‐MRI: dynamic contrast‐enhanced magnetic resonance imaging
HZ Hospital: Huizhou Central People's Hospital
LY Hospital: Linyi City People's Hospital
MRI: magnetic resonance imaging
PACS: picture archiving and communication system
PTR: the peritumoral region
ROC: receiver operating characteristics
ROI: region of interest
SYSU Hospital: the Fifth Affiliated Hospital of Sun Yat‐sen University
VOI: volumes of interest

## 1 Introduction

According to the International Agency for Research on Cancer, breast cancer is one of the most prevalent malignant tumors globally, particularly among women [1, 2]. This high incidence and mortality rate are partly attributable to the complexity and heterogeneity of breast cancer, which complicates treatment and prognosis [3, 4, 5]. Breast cancer can be classified into various molecular subtypes based on specific receptors, each exhibiting differences in behavior, prognosis, and treatment strategies. Identifying biomarkers through tissue biopsy or imaging is essential, but biopsies are invasive and constrained by tumor characteristics [6].

Magnetic resonance imaging (MRI), particularly dynamic contrast‐enhanced MRI (DCE‐MRI), is a noninvasive modality extensively applied in breast cancer diagnosis and assessment [7, 8, 9]. MRI offers high‐resolution and detailed information on lesion characteristics [10, 11]. However, manual interpretation of MRI to assess tumor heterogeneity remains challenging.

Radiomics, as an emerging interdisciplinary technology, extracts numerous quantitative features (such as shape, texture, and intensity) from medical images, offering new possibilities for personalized tumor diagnosis and treatment [12, 13]. Radiomics can capture the heterogeneity within tumors and analyze changes in their surrounding microenvironment [14]. Deep learning, particularly convolutional neural networks (CNNs), automates hierarchical feature extraction from raw imaging data, thereby overcoming the limitations of traditional radiomics, which relies on manual feature engineering [15]. Transfer learning further enhances this capability by leveraging pretrained models (e.g., those trained on ImageNet) and fine‐tuning them for medical tasks, thereby effectively addressing data scarcity issues [16].

In medical image analysis, the spatial representation of regions of interest (ROI) establishes fundamental feature extraction paradigms. Traditional radiomics predominantly relies on 3D volumetric analysis for manual texture quantification, whereas deep learning frameworks exhibit dimensional adaptability—effectively processing 2.5D inputs while preserving three‐dimensional contextual awareness at substantially reduced computational costs [17, 18]. Despite this advantage, the synergistic effects of ROI dimensionality (2D/2.5D/3D), peritumoral expansion levels (0–8 mm), and segmentation scenarios (ROI only vs. ROI original) remain inadequately characterized in breast cancer subtyping. Our study systematically delineates how these interdependent operational parameters collectively influence diagnostic performance, constructing a methodological framework that harmonizes radiomics‐inspired spatial principles with deep learning efficiency. This dual‐perspective investigation demonstrates that dimensional optimization enables high‐fidelity malignancy characterization while alleviating hardware constraints, thereby bridging domain‐specific radiomics knowledge with automated feature learning architectures.

This study evaluates the performance of deep learning models trained with transfer learning techniques by comparing different ROI dimensions (2D, 2.5D, 3D) and peritumoral expansion levels (0–8 mm) under two segmentation scenarios (ROI only and ROI original), aiming to distinguish luminal from nonluminal breast cancers using DCE‐MRI. Our study systematically delineates how these interdependent operational parameters collectively influence diagnostic performance, constructing a methodological framework that harmonizes radiomics‐inspired spatial principles with deep learning efficiency. This dual‐perspective investigation demonstrates that dimensional optimization enables high‐fidelity malignancy characterization while alleviating hardware constraints, thereby bridging domain‐specific radiomics knowledge with automated feature learning architectures.

## 2 Materials and Methods

### 2.1 Patients

**FIGURE 1:**
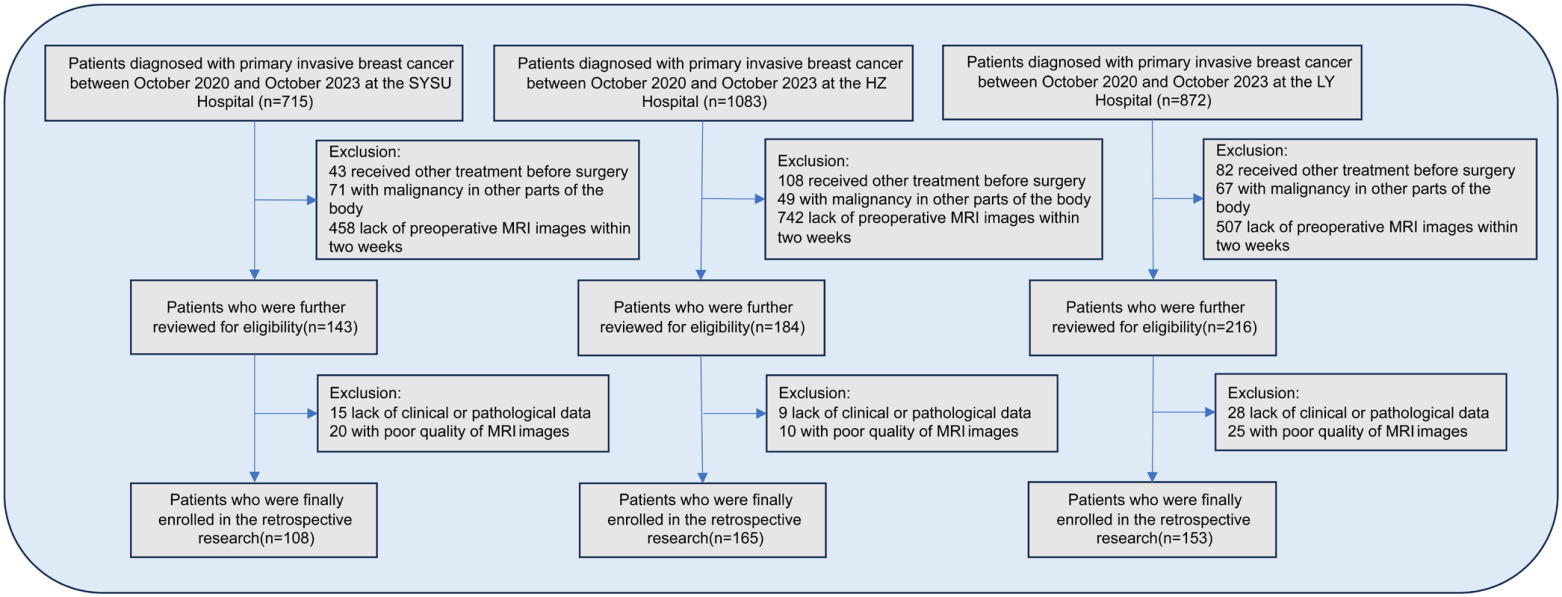
Flowchart of patient selection.

**FIGURE 2:**
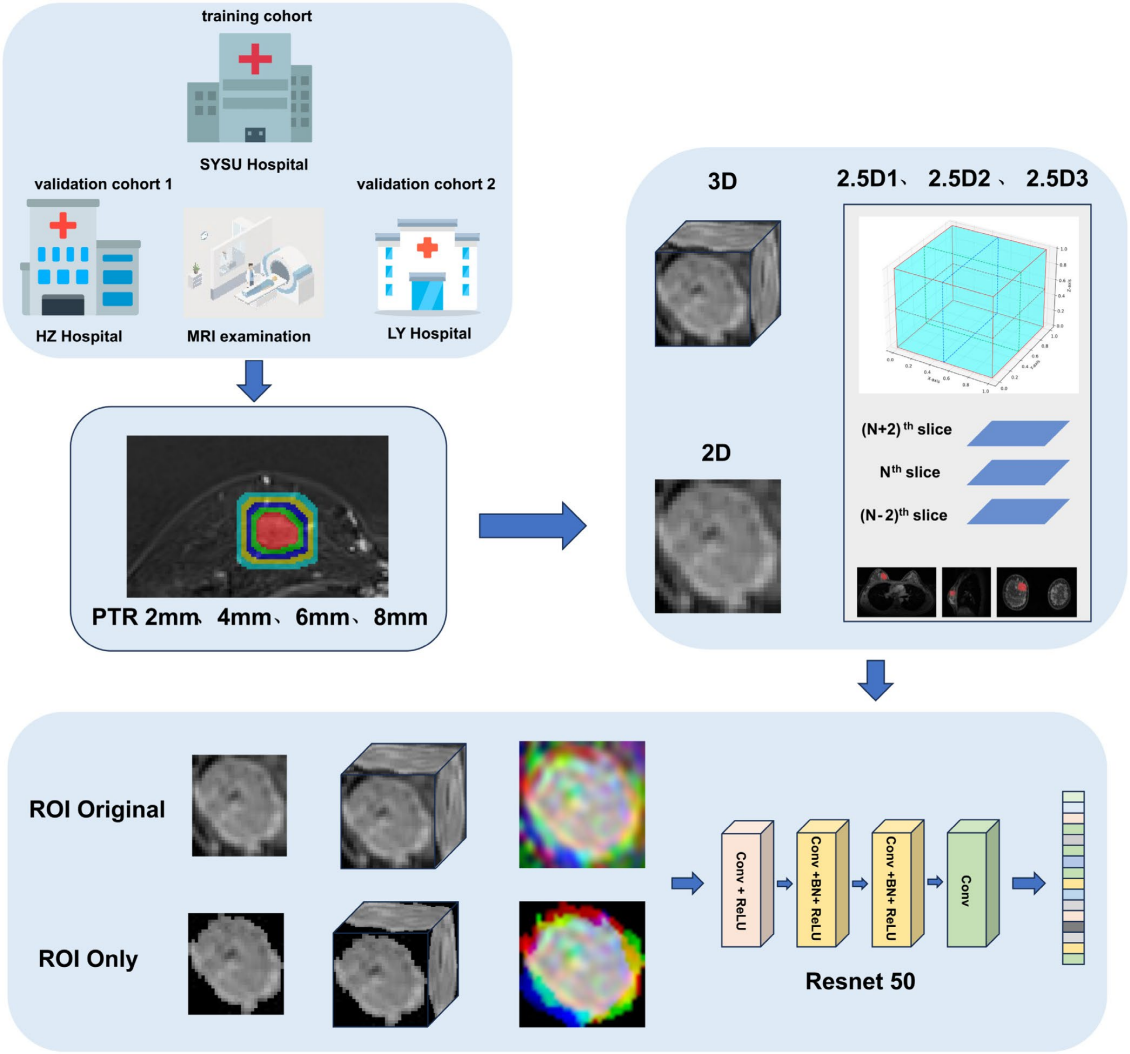
The study design and workflow of model development.

This study included patients diagnosed with primary invasive breast cancer between October 2020 and October 2023 at the SYSU Hospital, HZ Hospital, and LY Hospital. The inclusion criteria were as follows: (1) pathologically confirmed primary invasive breast cancer via surgery or biopsy; (2) patients with a solitary solid tumor; (3) underwent breast MRI, including DCE‐MRI, within 2 weeks prior to surgery or biopsy; and (4) without neoadjuvant chemotherapy or other interventions prior to imaging. The exclusion criteria were as follows: (1) received any form of treatment before imaging; (2) presence of malignancies in other locations; (3) incomplete clinical, MRI, or pathological data; (4) poor MRI image. 426 patients were ultimately included and divided into three cohorts (Figure 1): (1) training cohort, *n* = 108, from the SYSU Hospital (Zhuhai, China); (2) validation cohort 1, *n* = 165, from HZ Hospital (Huizhou, China); and (3) validation cohort 2, *n* = 153, from LY Hospital (Linyi, China). Table 1 summarizes the clinical characteristics of patients in the training and validation cohorts. Clinical features were collected for descriptive purposes only. These variables were not used as inputs to the deep learning models, which were trained and validated solely on DCE‐MRI data. The current study design is illustrated in Figure 2.

**TABLE 1:** Baseline table of the patients.

Variable	Group				*p* ^b^
	Overall, *N* = 426^a^	Train, *N* = 108^a^	Validation 1, *N* = 165^a^	Validation 2, *N* = 153^a^	
Age, years					0.11
Median (IQR)	48 (41, 54)	48 (44, 59)	48 (42, 54)	48 (39, 54)	
Mean (SD)	48.34 ± 10.44	50.43 ± 11.45	48.16 ± 9.14	47.07 ± 10.86	
Range	21, 80	28, 80	28, 72	21, 74	
Diameter, mm					0.106
Median (IQR)	23 (17, 31)	22.5 (15, 29)	24 (17, 37)	23 (17, 30)	
Mean (SD)	26.24 ± 14.29	22.31 ± 11.08	27.96 ± 15.06	26.44 ± 15.17	
Range	7, 109	8, 87	10, 109	7, 61	
Histology					0.194
Triple‐negative	46 (10.8)	9 (8.33)	22 (13.3)	15 (9.80)	
Luminal	307 (72.1)	78 (72.2)	123 (74.5)	106 (69.3)	
HER‐2	73 (17.1)	21 (19.4)	20 (12.1)	32 (20.9)	
Location, *n* (%)					0.195
Upper inner	110 (25.8)	32 (29.6)	41 (24.9)	37 (24.2)	
Lower inner	65 (15.3)	18 (16.7)	30 (18.18)	17 (11.1)	
Outer upper	168 (39.4)	36 (33.3)	59 (35.76)	73 (47.7)	
Outer lower	83 (19.5)	22 (20.7)	35 (21.21)	26 (17.0)	
BI‐RADS, *n* (%)					0.115
≤ 4b	70 (16.4)	20 (18.5)	25 (15.2)	25 (16.3)	
4c	107 (25.1)	21 (19.4)	35 (21.2)	51 (33.3)	
5	134 (31.5)	36 (33.3)	54 (32.7)	44 (28.8)	
6	115 (27.0)	31 (28.7)	51 (30.9)	33 (21.6)	
Grading, *n* (%)					0.597
1	56 (13.1)	10 (9.26)	23 (13.9)	23 (15.0)	
2	236 (55.4)	59 (54.6)	92 (55.8)	85 (55.6)	
3	134 (31.5)	39 (36.1)	50 (30.3)	45 (29.4)	

^a^ Median (IQR) or frequency (%).

^b^ Kruskal–Wallis rank sum test, Fisher's exact test, Pearson's chi‐squared test.

### 2.2 MRI Data Acquisition

All patients underwent breast DCE‐MRI examination within 2 weeks before surgery or biopsy. MRI scans were performed using different models of magnetic resonance imaging equipment at different hospitals, but all scans adhered to standardized protocols (details in Supporting Information S1). All patients' DCE images (identified at the phase of maximum lesion enhancement based on the DCE time‐signal intensity curve) were exported in “Dicom” format from the picture archiving and communication system (PACS). Our study prioritized the peak enhancement phase (determined by time–intensity curves) for DCE‐MRI analysis, as it optimally differentiates lesion vascularity from surrounding tissues and correlates with histopathological angiogenesis markers [19, 20]. While acknowledging the theoretical advantages of multiphase/4D analysis [21], we adopted the clinically pragmatic single‐phase approach to balance diagnostic relevance and workflow efficiency.

### 2.3 MRI Imaging Processing

The DCE‐MRI images in this study were obtained from different medical centers and scanning equipment. To ensure image quality and data consistency, preprocessing steps are crucial. First, the *z*‐score normalization method was applied to standardize the signal intensity of the images, ensuring consistency. Then, the images were resampled to a voxel size of 1.0 × 1.0 × 1.0 mm [3] to ensure consistent spatial resolution during analysis. Finally, to further improve image quality, the N4 bias field correction method was applied to correct non‐uniform magnetic field effects in the images.

### 2.4 Tumor Segmentation

Tumor segmentation was performed by two breast surgeons and radiologists with 30 and 15 years of experience, respectively. Blinded to pathological information, they manually delineated each layer of the lesion along its edge, ultimately generating a three‐dimensional volume of interest (VOI). All segmentations were subsequently reviewed by a third senior radiologist (25 years of experience in breast imaging) to resolve discrepancies. This rigorous annotation protocol, incorporating multi‐expert verification, ensured the stability of ROI delineations. Such methodological robustness provided a solid foundation for subsequent comparative analyses across different processing configurations, including two segmentation scenarios (ROI only vs. ROI original), peritumoral expansion levels (0–8 mm), and multidimensional ROI representations (2D/2.5D/3D). Following manual tumor segmentation, to capture radiomics features of the peritumoral region, we used the “SimpleITK” package in Python 3.12.4 to automatically extend outward from the lesion boundary by 2, 4, 6, and 8 mm, generating volumes of interest (VOI) for the peritumoral region (PTR). If the expanded peritumoral region exceeded the breast parenchyma, the extraneous parts were manually removed.

### 2.5 2.5D ROI Extraction Methods

**FIGURE 3:**
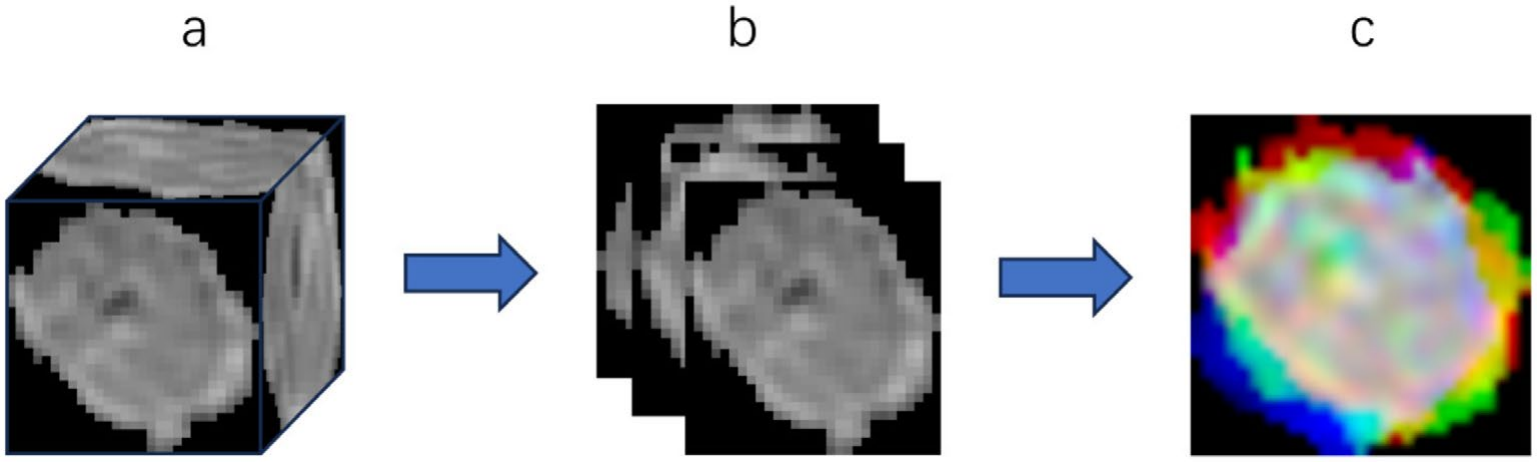
The processing steps for the 2.5D1 (ROI only) method. (a) the minimum bounding cube of ROI only, (b) the three primary slices of the minimum bounding cube, (c) multichannel image.

2.5D ROI refers to a method of extending and comprehensively analyzing a sequence of 2D images along the third dimension (usually the slice direction). Three 2.5D strategies were implemented to approximate 3D tumor context while retaining computational efficiency (Appendix Figure S1): 2.5D1: Extracts the three orthogonal planes (axial, sagittal, and coronal) intersecting at the tumor centroid, capturing multidirectional features; 2.5D2: Aggregates the central tumor slice with its immediate adjacent slices (±2 slice) to encode local spatial continuity; 2.5D3: Generates maximum ROI area across multiview planes (e.g., sagittal, coronal, axial) to highlight dominant tumor characteristics (Figure 3).

These methods enable 3D‐aware feature learning without requiring full 3D convolutions, aligning with clinical workflows where 3D reconstruction is often impractical. The detailed steps and processes of these three methods are illustrated in Supporting Information S1. By employing these methods, each utilizing different aspects of 2.5D imaging, researchers can leverage a combination of comprehensive spatial information, contextual details, and multidirectional perspectives to enhance the performance of deep learning models in medical image analysis.

### 2.6 Definition of ROI Only

ROI only: The tumor region was precisely segmented, excluding all extratumoral tissue. This mimics radiomics workflows where only intratumoral features are analyzed; ROI original: The tumor region was retained with its native surrounding tissue, allowing the model to learn peritumoral microenvironment features (e.g., angiogenesis, immune infiltration).

To assess the impact of tumor boundary definition, we compared two segmentation strategies: [1] ROI only (isolated tumor region) vs. [2] ROI original (tumor with native peritumoral context), evaluating their differential effects on deep learning model performance. The implementation pipelines for 2D (slicewise), 2.5D (multi‐slice), and 3D (volumetric) processing under these protocols are detailed in Appendix Figures S2–S4.

### 2.7 Model Construction and Assessment

To train the deep convolutional neural network (CNN) model, we employed the ResNet‐50 architecture, loading pretrained weights from ImageNet. ResNet‐50 is a deep learning model trained on the ILSVRC‐2012 data set [22]. This model has been reported to perform exceptionally well in medical image processing, with its deep feature learning capabilities and effective transfer learning applications, making it widely used in medical image classification, detection, and segmentation tasks. Aggressive data augmentation techniques, such as random cropping, horizontal flip, and vertical flip, were employed in model construction. The performance of all established models was evaluated using receiver operating characteristic (ROC) analysis, with the area under the ROC curve (AUC) calculated and compared using the DeLong test. Additionally, the accuracy, sensitivity, and specificity of the predictions were measured to comprehensively assess the diagnostic performance of the model.

### 2.8 Statistical Analysis

This study employed the gtsummary package [23] in R software (version 4.2.3) for statistical analysis of baseline data. Continuous variables were described using median (interquartile range), mean ± standard deviation, and range, while categorical variables were expressed as frequency and percentage. Comparisons of continuous variables between groups were performed using the Kruskal–Wallis rank‐sum test, whereas categorical variables were compared using Pearson's chi‐squared test or Fisher's exact test. Comparisons of the area under the curve (AUC) were performed using the DeLong test. In all analyses, a *p*‐value of less than 0.05 was considered statistically significant.

### 2.9 Data Set Description and Clinical Characteristics

Table 1 summarizes the baseline characteristics of all patients. These analyses were performed solely to characterize the data set and confirm balanced distributions across cohorts (all *p* > 0.05). No clinical features were integrated into the deep learning models, which relied exclusively on imaging data. This study enrolled a total of 426 patients with invasive breast cancer, divided into a training cohort, validation cohort 1, and validation cohort 2 (108, 165, and 153 patients, respectively). All patients were diagnosed with invasive breast cancer. The mean age of the entire cohort was 48.34 years (standard deviation: 10.44), with a median age of 48 years (interquartile range: 41–54). There were no statistically significant differences in age distribution among the training, validation 1, and validation 2 cohorts (*p* > 0.05). The cohort included 307 patients with luminal‐type breast cancer, 46 with triple‐negative breast cancer, and 73 with Her2‐type breast cancer. Luminal‐type accounted for 72.1%, whereas nonluminal types accounted for 27.9%. The mean maximum tumor diameter was 26.24 mm, with a median of 23 mm. The distribution of tumor locations was as follows: upper inner quadrant (25.8%), lower inner quadrant (15.3%), upper outer quadrant (39.4%), and lower outer quadrant (19.5%). There were no statistically significant differences between groups (*p* = 0.195). BI‐RADS classification showed that 16.4% of patients were ≤ 4b, 25.1% were 4c, 31.5% were grade 5, and 27.0% were grade 6. There were no statistically significant differences in BI‐RADS distribution between groups (*p* = 0.115). Histological grading distribution was as follows: grade 1 (13.1%), grade 2 (35.9%), and grade 3 (51.1%). There were no significant differences between groups (*p* = 0.597).

## 3 Results

### 3.1 Comparison of the DCNN Models (ROI Only)

**FIGURE 4:**
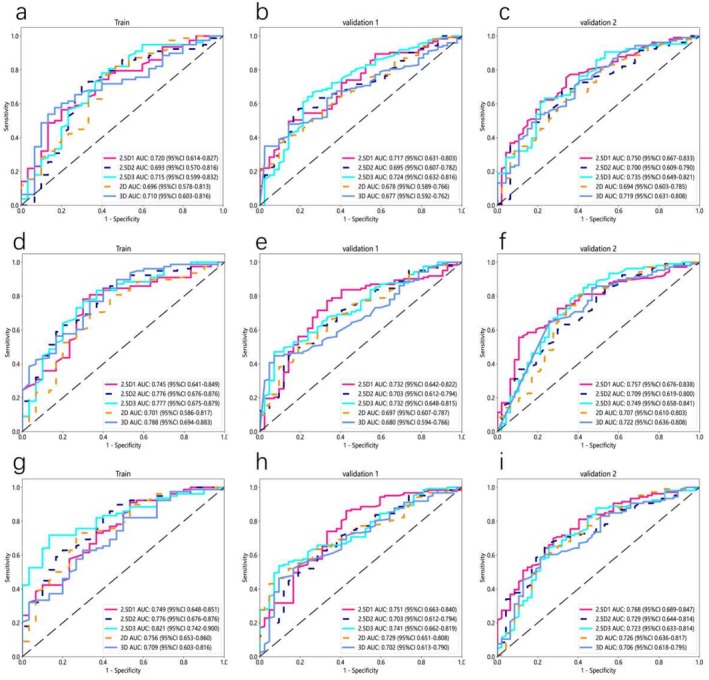
ROC curve of deep learning models based on 2D, 2.5D1, 2.5D2, 2.5D3, and 3D techniques for PTR measurements at 0 mm (a–c), 2 mm (d–f), and 4 mm (g–i).

**FIGURE 5:**
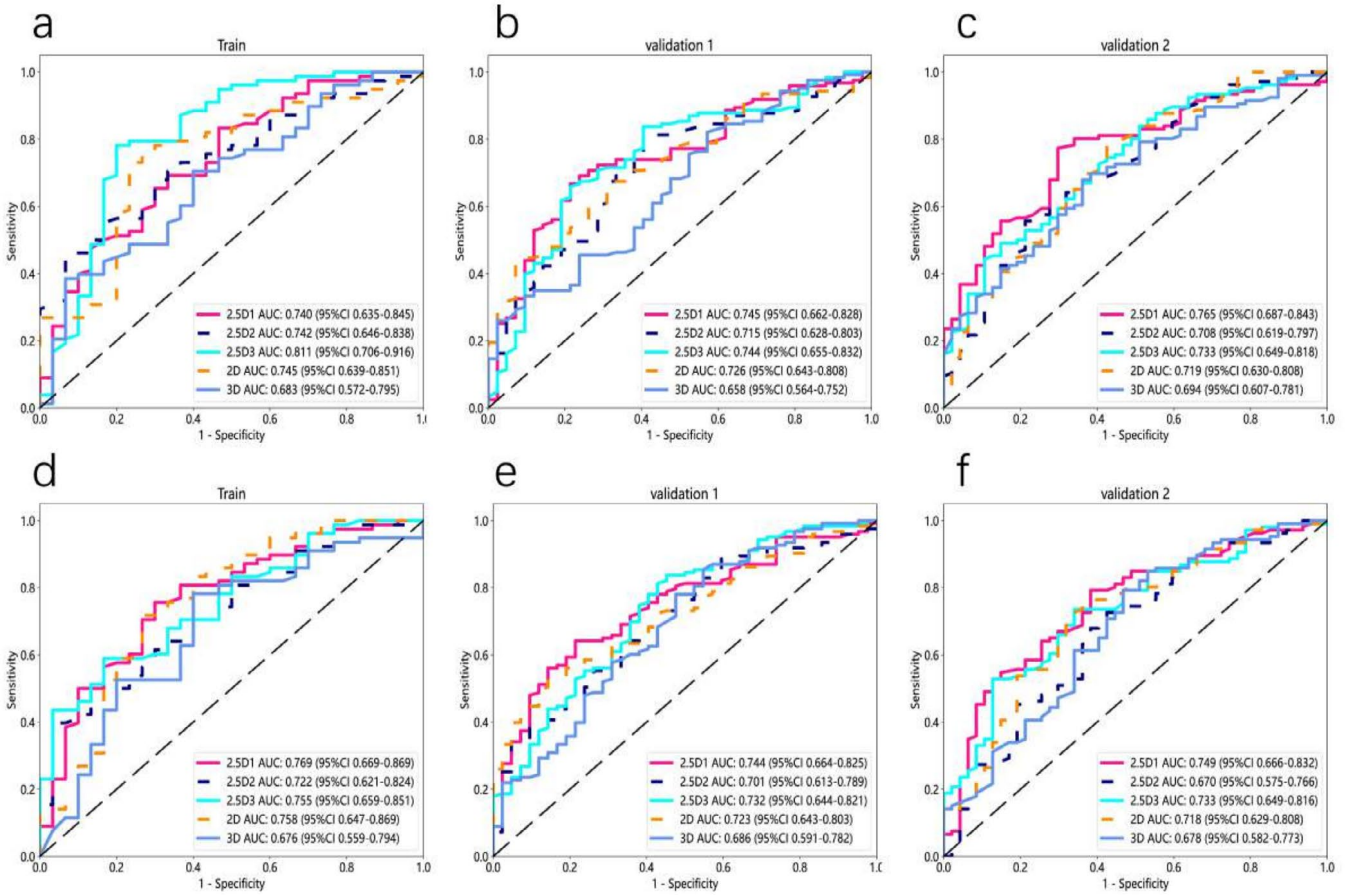
ROC curve of deep learning models based on 2D, 2.5D1, 2.5D2, 2.5D3, and 3D techniques for PTR measurements at 6 mm (a, b, c), and 8 mm (d, e, f).

The following (Figures 4 and 5) are the results of a study focusing on the precise segmentation of the region of interest (ROI) only, in which only the ROI was retained during model construction, excluding background and normal tissue using a deep neural network model. Without peritumoral expansion (Figure 4a–c), the 2.5D1‐based model demonstrated a higher AUC value and consistent performance across different data sets. In the training cohort, the AUC was 0.720 (95% CI 0.614–0.827); in validation cohort 1, the AUC was 0.717 (95% CI 0.631–0.803); and in validation cohort 2, the AUC was 0.750 (95% CI 0.667–0.833). In contrast, the 2D‐based model demonstrated an AUC of 0.696 (95% CI 0.578–0.813) in the training cohort, 0.678 (95% CI 0.589–0.766) in validation cohort 1, and 0.694 (95% CI 0.603–0.785) in validation cohort 2. The 3D‐based model exhibited an AUC of 0.720 (95% CI 0.614–0.827) in the training cohort, 0.717 (95% CI 0.631–0.803) in validation cohort 1, and 0.750 (95% CI 0.667–0.833) in validation cohort 2.

Among the 2.5D models, the performance of the 2.5D1‐based deep learning model was superior to that of the 2.5D2‐based and 2.5D3‐based models. Specifically, the 2.5D2‐based model exhibited an AUC of 0.693 (95% CI 0.570–0.816) in the training cohort, 0.695 (95% CI 0.607–0.782) in validation cohort 1, and 0.700 (95% CI 0.609–0.790) in validation cohort 2. The 2.5D3‐based model demonstrated an AUC of 0.715 (95% CI 0.599–0.832) in the training cohort, 0.724 (95% CI 0.632–0.816) in validation cohort 1, and 0.735 (95% CI 0.649–0.821) in validation cohort 2.

After expanding the peritumoral region, we found that when the region of interest (ROI) was extended outward by 4 mm (Figure 4g–i), the 2D‐based, 2.5D‐based, and 3D‐based models all demonstrated optimal performance, with the 2.5D1‐based model performing the best. Specifically, in the training cohort, the 2.5D1‐based model achieved an AUC of 0.749 (95% CI 0.648–0.851); in validation cohort 1, the AUC was 0.751 (95% CI 0.663–0.840); and in validation cohort 2, the AUC was 0.768 (95% CI 0.689–0.847). In contrast, the 2D‐based model demonstrated an AUC of 0.756 (95% CI 0.653–0.860) in the training cohort, 0.729 (95% CI 0.651–0.808) in validation cohort 1, and 0.726 (95% CI 0.636–0.817) in validation cohort 2. The 3D‐based model exhibited an AUC of 0.709 (95% CI 0.603–0.816) in the training cohort, 0.702 (95% CI 0.613–0.790) in validation cohort 1, and 0.706 (95% CI 0.618–0.795) in validation cohort 2.

Among the 2.5D‐based models, the performance of the 2.5D1‐based deep learning model was superior to that of the 2.5D2‐based and 2.5D3‐based models. Specifically, the 2.5D2‐based model demonstrated an AUC of 0.776 (95% CI 0.676–0.876) in the training cohort, 0.703 (95% CI 0.612–0.794) in validation cohort 1, and 0.729 (95% CI 0.644–0.814) in validation cohort 2. Additionally, the 2.5D3‐based model achieved an AUC of 0.821 (95% CI 0.742–0.900) in the training cohort, 0.741 (95% CI 0.662–0.819) in validation cohort 1, and 0.723 (95% CI 0.633–0.814) in validation cohort 2. Diagnostic performance metrics, including AUC, sensitivity, specificity, and accuracy, are comprehensively compared in Tables 2 and 3.

**TABLE 2:** Diagnostic performance of 2D, 2.5D, and 3D deep convolutional neural networks (DCNN) models with peritumoral region (PTR) analysis (0 mm, 2 mm, 4 mm; ROI only).

		0 mm					2 mm					4 mm				
		2.5D1	2.5D2	2.5D3	2D	3D	2.5D1	2.5D2	2.5D3	2D	3D	2.5D1	2.5D2	2.5D3	2D	3D
Train	AUC	0.720	0.693	0.715	0.696	0.710	0.745	0.776	0.777	0.701	0.788	0.749	0.776	0.821	0.756	0.709
	95%CI	0.614–0.827	0.570–0.816	0.599–0.832	0.578–0.813	0.603–0.816	0.641–0.849	0.676–0.876	0.675–0.879	0.586–0.817	0.694–0.883	0.648–0.851	0.676–0.876	0.742–0.900	0.653–0.860	0.603–0.816
	Sensitivity	0.708	0.705	0.713	0.756	0.564	0.769	0.821	0.756	0.795	0.75	0.897	0.722	0.705	0.718	0.615
	Specificity	0.667	0.70	0.714	0.600	0.833	0.700	0.600	0.700	0.567	0.778	0.467	0.741	0.867	0.733	0.700
	Accuracy	0.697	0.704	0.713	0.713	0.639	0.750	0.759	0.741	0.731	0.758	0.778	0.727	0.750	0.722	0.639
Validation1	AUC	0.717	0.695	0.724	0.678	0.677	0.732	0.703	0.732	0.697	0.680	0.751	0.703	0.741	0.729	0.702
	95% CI	0.631–0.803	0.607–0.782	0.632–0.816	0.589–0.766	0.592–0.762	0.642–0.822	0.612–0.794	0.648–0.815	0.607–0.787	0.594–0.766	0.663–0.840	0.612–0.794	0.662–0.819	0.651–0.808	0.613–0.790
	Sensitivity	0.480	0.561	0.659	0.382	0.472	0.756	0.699	0.463	0.504	0.439	0.854	0.881	0.520	0.431	0.455
	Specificity	0.857	0.810	0.738	0.881	0.857	0.667	0.619	0.929	0.810	0.929	0.571	0.514	0.905	0.929	0.905
	Accuracy	0.576	0.624	0.679	0.509	0.570	0.733	0.679	0.582	0.582	0.564	0.782	0.787	0.618	0.558	0.570
Validation2	AUC	0.750	0.700	0.735	0.694	0.719	0.757	0.709	0.749	0.707	0.722	0.768	0.729	0.723	0.726	0.706
	95% CI	0.667–0.833	0.609–0.790	0.649–0.821	0.603–0.785	0.631–0.808	0.676–0.838	0.619–0.800	0.658–0.841	0.610–0.803	0.636–0.808	0.689–0.847	0.644–0.814	0.633–0.814	0.636–0.817	0.618–0.795
	Sensitivity	0.736	0.673	0.623	0.660	0.726	0.547	0.811	0.840	0.792	0.972	0.689	0.642	0.628	0.694	0.575
	Specificity	0.660	0.657	0.766	0.660	0.596	0.894	0.511	0.574	0.596	0.481	0.723	0.766	0.8	0.704	0.787
	Accuracy	0.712	0.669	0.667	0.660	0.686	0.654	0.719	0.758	0.732	0.838	0.699	0.680	0.676	0.697	0.641

**TABLE 3:** Diagnostic performance of 2D, 2.5D, and 3D deep convolutional neural networks (DCNN) models with peritumoral region (PTR) analysis (6 mm, 8 mm; ROI only).

		6 mm					8 mm				
		2.5D1	2.5D2	2.5D3	2D	3D	2.5D1	2.5D2	2.5D3	2D	3D
Train	AUC	0.740	0.742	0.811	0.745	0.683	0.769	0.722	0.755	0.758	0.676
	95% CI	0.635–0.845	0.646–0.838	0.706–0.916	0.639–0.851	0.572–0.795	0.669–0.869	0.621–0.824	0.659–0.851	0.647–0.869	0.559–0.794
	Sensitivity	0.821	0.449	0.769	0.705	0.372	0.744	0.385	0.577	0.705	0.769
	Specificity	0.533	0.933	0.800	0.767	0.933	0.700	0.967	0.833	0.733	0.600
	Accuracy	0.741	0.583	0.778	0.722	0.528	0.731	0.546	0.648	0.713	0.722
Validation1	AUC	0.745	0.715	0.744	0.726	0.658	0.744	0.701	0.732	0.723	0.686
	95% CI	0.662–0.828	0.628–0.803	0.655–0.832	0.643–0.808	0.564–0.752	0.664–0.825	0.613–0.789	0.644–0.821	0.643–0.803	0.591–0.782
	Sensitivity	0.683	0.789	0.642	0.423	0.805	0.634	0.333	0.829	0.520	0.837
	Specificity	0.762	0.595	0.786	0.929	0.429	0.786	0.952	0.548	0.857	0.452
	Accuracy	0.703	0.739	0.679	0.552	0.709	0.673	0.491	0.758	0.606	0.739
Validation2	AUC	0.765	0.708	0.733	0.719	0.694	0.749	0.670	0.733	0.718	0.678
	95% CI	0.687–0.843	0.619–0.797	0.649–0.818	0.630–0.808	0.607–0.781	0.666–0.832	0.575–0.766	0.649–0.816	0.629–0.808	0.582–0.773
	Sensitivity	0.764	0.538	0.481	0.774	0.670	0.783	0.717	0.519	0.535	0.783
	Specificity	0.702	0.787	0.851	0.574	0.638	0.617	0.574	0.872	0.829	0.532
	Accuracy	0.745	0.614	0.595	0.712	0.660	0.732	0.673	0.627	0.61	0.706

Overall, the performance of the 2.5D‐based deep learning models was superior, as evidenced by narrow confidence intervals for the AUC values, indicating stable performance. The 2.5D1 model (PTR 4 mm) demonstrated the best performance.

### 3.2 Comparison of the DCNN Models (ROI Original)

**FIGURE 6:**
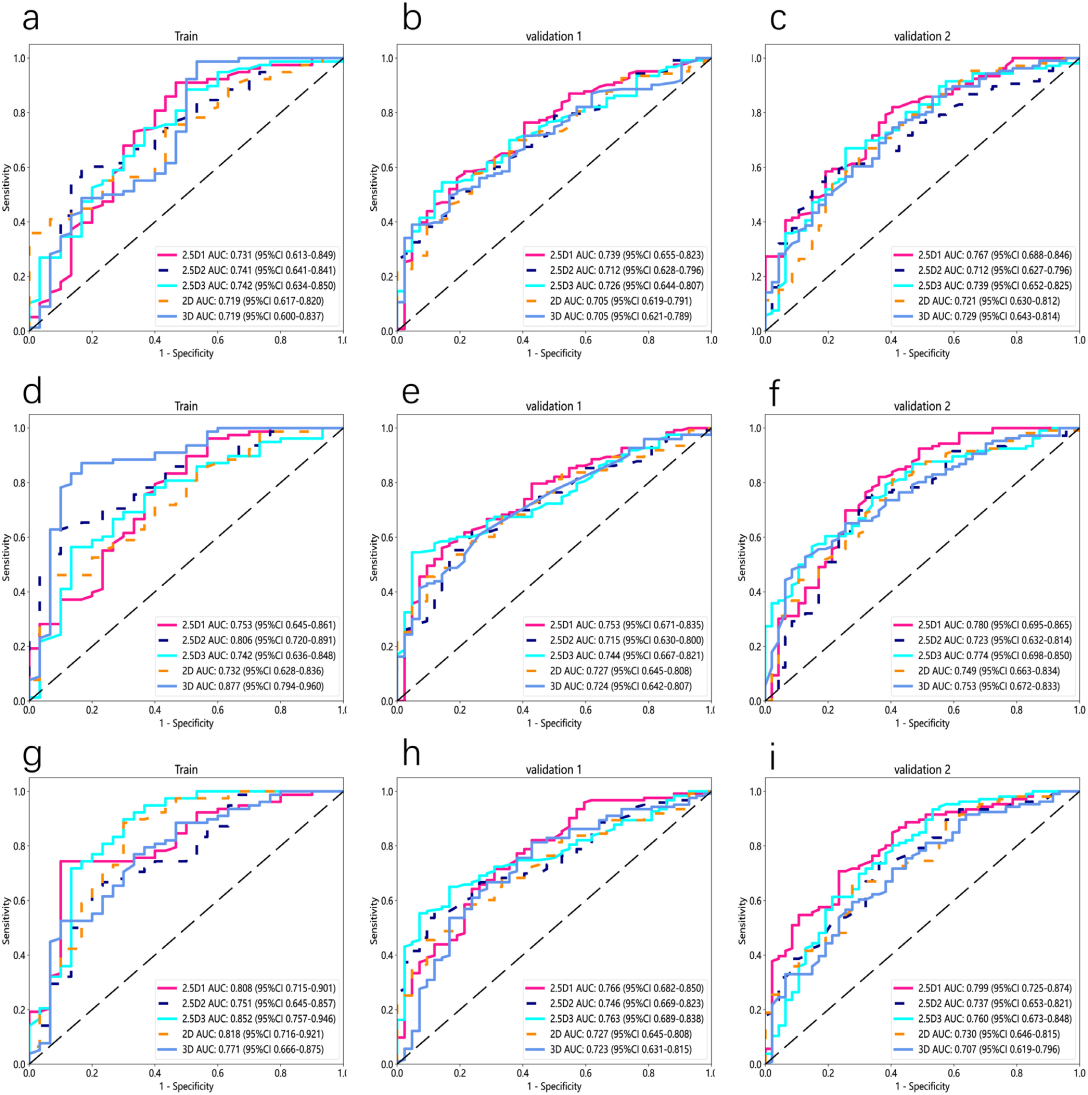
ROC curve of deep learning models based on 2D, 2.5D1, 2.5D2, 2.5D3, and 3D techniques for PTR measurements at 0 mm (a–c), 2 mm (d–f), and 4 mm (g–i).

**FIGURE 7:**
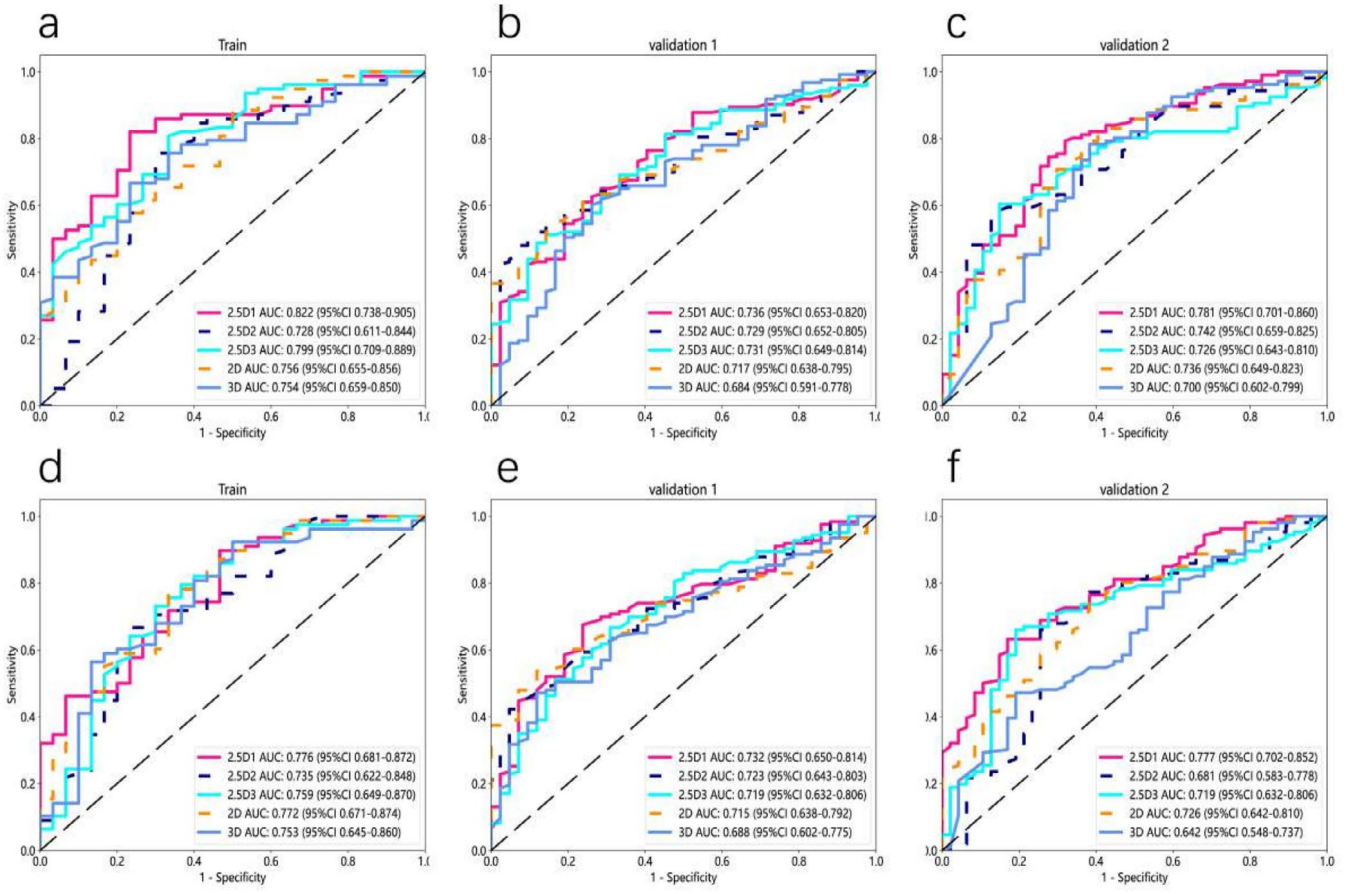
ROC curve of deep learning models based on 2D, 2.5D1, 2.5D2, 2.5D3, and 3D techniques for PTR measurements at 6 mm (a, b, c), and 8 mm (d, e, f).

The following (Figures 6 and 7) present the results of the deep neural network models retaining the background and normal tissue. Without peritumoral expansion (Figure 6a–c), the 2.5D1 model exhibited high and consistent AUC values: 0.731 (95% CI 0.613–0.849) in the training cohort, 0.739 (95% CI 0.655–0.823) in Validation Cohort 1, and 0.767 (95% CI 0.688–0.846) in Validation Cohort 2. In contrast, the 2D model had AUC values of 0.719 (95% CI 0.617–0.820) in the training cohort, 0.705 (95% CI 0.619–0.791) in Validation Cohort 1, and 0.721 (95% CI 0.630–0.812) in Validation Cohort 2. Similarly, the 3D model had AUC values of 0.719 (95% CI 0.600–0.837) in the training cohort, 0.705 (95% CI 0.621–0.789) in Validation Cohort 1, and 0.729 (95% CI 0.643–0.814) in Validation Cohort 2.

Among the 2.5D‐based models, the 2.5D3‐based model outperformed the 2.5D2‐based model. Specifically, the 2.5D2‐based model had AUC values of 0.741 (95% CI 0.641–0.841) in the training cohort, 0.712 (95% CI 0.628–0.796) in Validation Cohort 1, and 0.712 (95% CI 0.627–0.796) in Validation Cohort 2. The 2.5D3‐based model had AUC values of 0.742 (95% CI 0.634–0.850) in the training cohort, 0.726 (95% CI 0.644–0.807) in Validation Cohort 1, and 0.739 (95% CI 0.652–0.825) in Validation Cohort 2.

After peritumoral expansion, it was found that when the region of interest was expanded outward by 4 mm (Figure 6g–i), all models demonstrated optimal performance, with the 2.5D1‐based model performing the best. In the training cohort, the AUC was 0.808 (95% CI 0.715–0.901); in validation cohort 1, the AUC was 0.766 (95% CI 0.682–0.850), and in validation cohort 2, the AUC was 0.799 (95% CI 0.725–0.874). In comparison, the 2D‐based model had an AUC of 0.818 (95% CI 0.716–0.921) in the training cohort, 0.727 (95% CI 0.645–0.808) in validation cohort 1, and 0.730 (95% CI 0.646–0.815) in validation cohort 2; the 3D model had an AUC of 0.771 (95% CI 0.666–0.875) in the training cohort, 0.723 (95% CI 0.631–0.815) in validation cohort 1, and 0.707 (95% CI 0.619–0.796) in validation cohort 2.

In the 2.5D‐based models, the performance of the 2.5D3‐based model was superior to the 2.5D2‐based model. Specifically, the 2.5D2‐based model had an AUC of 0.751 (95% CI 0.645–0.857) in the training cohort, 0.746 (95% CI 0.669–0.823) in validation cohort 1, and 0.737 (95% CI 0.653–0.821) in validation cohort 2. The 2.5D3‐based model, on the other hand, achieved an AUC of 0.852 (95% CI 0.757–0.946) in the training cohort, 0.763 (95% CI 0.689–0.838) in validation cohort 1, and 0.760 (95% CI 0.673–0.848) in validation cohort 2. Diagnostic performance metrics, including AUC, sensitivity, specificity, and accuracy, are comprehensively compared in Tables 4 and 5.

**TABLE 4:** Diagnostic performance of 2D, 2.5D, and 3D deep convolutional neural networks (DCNN) models with peritumoral region (PTR) analysis (0 mm, 2 mm, 4 mm; ROI original).

		0 mm					2 mm					4 mm				
		2.5D1	2.5D2	2.5D3	2D	3D	2.5D1	2.5D2	2.5D3	2D	3D	2.5D1	2.5D2	2.5D3	2D	3D
Train	AUC	0.739	0.741	0.742	0.719	0.719	0.753	0.806	0.742	0.732	0.877	0.808	0.751	0.852	0.818	0.771
	95% CI	0.655–0.823	0.641–0.841	0.634–0.850	0.617–0.820	0.600–0.837	0.645–0.861	0.720–0.891	0.636–0.848	0.628–0.836	0.794–0.960	0.715–0.901	0.645–0.857	0.757–0.946	0.716–0.921	0.666–0.875
	Sensitivity	0.577	0.577	0.872	0.346	0.974	0.821	0.615	0.526	0.752	0.859	0.731	0.628	0.885	0.872	0.756
	Specificity	0.786	0.833	0.500	1.000	0.467	0.567	0.900	0.867	0.629	0.833	0.900	0.800	0.700	0.700	0.667
	Accuracy	0.630	0.648	0.769	0.528	0.833	0.750	0.694	0.620	0.721	0.852	0.778	0.676	0.833	0.824	0.731
Validation1	AUC	0.731	0.712	0.726	0.705	0.705	0.753	0.715	0.744	0.727	0.724	0.766	0.746	0.763	0.746	0.723
	95% CI	0.613–0.849	0.628–0.796	0.644–0.807	0.619–0.791	0.621–0.789	0.671–0.835	0.630–0.800	0.667–0.821	0.645–0,808	0.642–0.807	0.682–0.850	0.669–0.823	0.689–0.838	0.669–0.822	0.631–0.815
	Sensitivity	0.885	0.569	0.537	0.569	0.382	0.553	0.528	0.537	0.569	0.537	0.707	0.553	0.634	0.415	0.805
	Specificity	0.533	0.762	0.857	0.762	0.952	0.857	0.833	0.952	0.786	0.786	0.690	0.881	0.833	0.976	0.571
	Accuracy	0.787	0.618	0.618	0.618	0.527	0.630	0.606	0.642	0.624	0.600	0.703	0.636	0.685	0.558	0.745
Validation2	AUC	0.767	0.712	0.739	0.721	0.729	0.780	0.723	0,774	0.749	0.753	0.799	0.737	0.760	0.730	0.707
	95% CI	0.688–0.846	0.627–0.796	0.652–0.825	0.630–0.812	0.643–0.814	0.695–0.865	0.632–0.814	0.698–0.850	0.663–0.834	0.672–0.833	0.725–0.874	0.653–0.821	0.673–0.848	0.646–0.815	0.619–0.796
	Sensitivity	0.802	0.528	0.660	0.632	0.726	0.811	0.736	0.557	0.802	0.491	0.689	0.726	0.925	0.651	0.575
	Specificity	0.596	0.851	0.745	0.702	0.596	0.638	0.681	0.851	0.596	0.872	0.766	0.638	0.468	0.723	0.723
	Accuracy	0.739	0.627	0.686	0.654	0.686	0.758	0.719	0.647	0.739	0.608	0.712	0.699	0.784	0.673	0.621

**TABLE 5:** Diagnostic performance of 2D, 2.5D, and 3D deep convolutional neural networks (DCNN) models with peritumoral region (PTR) analysis (6 mm, 8 mm; ROI original).

		6 mm					8 mm				
		2.5D1	2.5D2	2.5D3	2D	3D	2.5D1	2.5D2	2.5D3	2D	3D
Train	AUC	0.822	0.728	0.799	0.756	0.754	0.776	0.735	0.759	0.772	0.753
	95% CI	0.738–0.905	0.611–0.844	0.709–0.889	0.655–0.856	0.659–0.850	0.681–0.872	0.622–0.848	0.649–0.870	0.671–0.874	0.645–0.860
	Sensitivity	0.808	0.744	0.795	0.872	0.654	0.885	0.654	0.718	0.769	0.551
	Specificity	0.767	0.700	0.667	0.500	0.767	0.533	0.767	0.700	0.667	0.867
	accuracy	0.796	0.731	0.759	0.769	0.685	0.787	0.685	0.862	0.741	0.639
Validation1	AUC	0.736	0.729	0.731	0.717	0.684	0.723	0.723	0.719	0.715	0.688
	95% CI	0.653–0.820	0.652–0.805	0.649–0.814	0.638–0.795	0.591–0.778	0.650–0.814	0.643–0.803	0.632–0.806	0.638–0.792	0.602–0.775
	Sensitivity	0.602	0.504	0.504	0.545	0.593	0.667	0.537	0.659	0.528	0.463
	Specificity	0.762	0.905	0.857	0.833	0.738	0.762	0.833	0.690	0.881	0.881
	Accuracy	0.642	0.606	0.594	0.618	0.630	0.691	0.612	0.667	0.618	0.570
Validation2	AUC	0.781	0.742	0.726	0.736	0.700	0.777	0.681	0.719	0.726	0.642
	95% CI	0.701–0.860	0.659–0.825	0.643–0.810	0.649–0.823	0.602–0.799	0.702–0.852	0.583–0.778	0.632–0.806	0.642–0.810	0.548–0.737
	Sensitivity	0.774	0.575	0.594	0.774	0.774	0.613	0.651	0.651	0.736	0.453
	Specificity	0.681	0.851	0.851	0.638	0.617	0.830	0.745	0.809	0.617	0.809
	Accuracy	0.745	0.660	0.673	0.732	0.725	0.680	0.680	0.699	0.699	0.562

Overall, when retaining background and normal tissue, the performance of 2D‐based, 2.5D‐based, and 3D‐based deep learning models improved compared to the precise segmentation of the tumor region (ROI only), with the best performance observed at a 4 mm peritumoral expansion. Among all models, the 2.5D1‐based model demonstrated the most stable and superior performance under this scenario, with AUC of 0.766 (sensitivity 0.731, specificity 0.900, accuracy 0.778) in the training cohort, 0.766 (sensitivity 0.707, specificity 0.690, accuracy 0.703) in validation cohort 1, and 0.799 (sensitivity 0.689, specificity 0.766, accuracy 0.712) in validation cohort 2.

### 3.3 Performance of the Predictive Model

**FIGURE 8:**
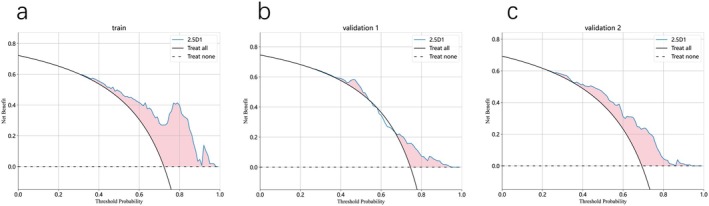
The decision curves of the 2.5D1 (ROI original) deep learning models for PTR 4 mm in the training cohort (a), validation cohort 1 (b), and validation cohort 2 (c).

We conducted decision curve analysis (DCA) to evaluate the clinical utility of the 2.5D1‐based model (PTR 4 mm) in the training cohort, validation cohort 1, and validation cohort 2 (Figure 8a–c). The 2.5D1 model demonstrated varying clinical utility across different threshold ranges. At low thresholds (0–0.4), the model exhibited superior net benefit, indicating optimal performance for initial screening scenarios with lenient treatment criteria. This range enables effective identification of a larger proportion of high‐risk patients requiring therapeutic intervention. When transitioning to moderate thresholds (0.4–0.7), although the net benefit showed gradual attenuation, the model maintained statistically significant advantages over the “Treat None” strategy. This intermediate range appears suitable for clinical decision‐making requiring balanced sensitivity and specificity, particularly when optimizing treatment allocation under moderately stringent criteria. Notably, the model's clinical value diminished substantially at high thresholds (0.7–1), where net benefit approached parity with the “Treat None” approach. This performance limitation suggests reduced effectiveness in ultra‐strict screening environments, necessitating complementary diagnostic modalities such as advanced imaging or histopathological confirmation to ensure comprehensive patient evaluation.

Clinical implementation recommendations propose preferential use in low‐to‐moderate threshold settings for early diagnostic applications and preventive interventions. However, in high‐threshold clinical scenarios demanding exceptional specificity, the current model architecture shows insufficient standalone performance to replace conventional screening protocols. Future optimization should focus on algorithmic enhancements or multimodal diagnostic integration to improve high‐threshold reliability for critical patient stratification.

**FIGURE 9:**
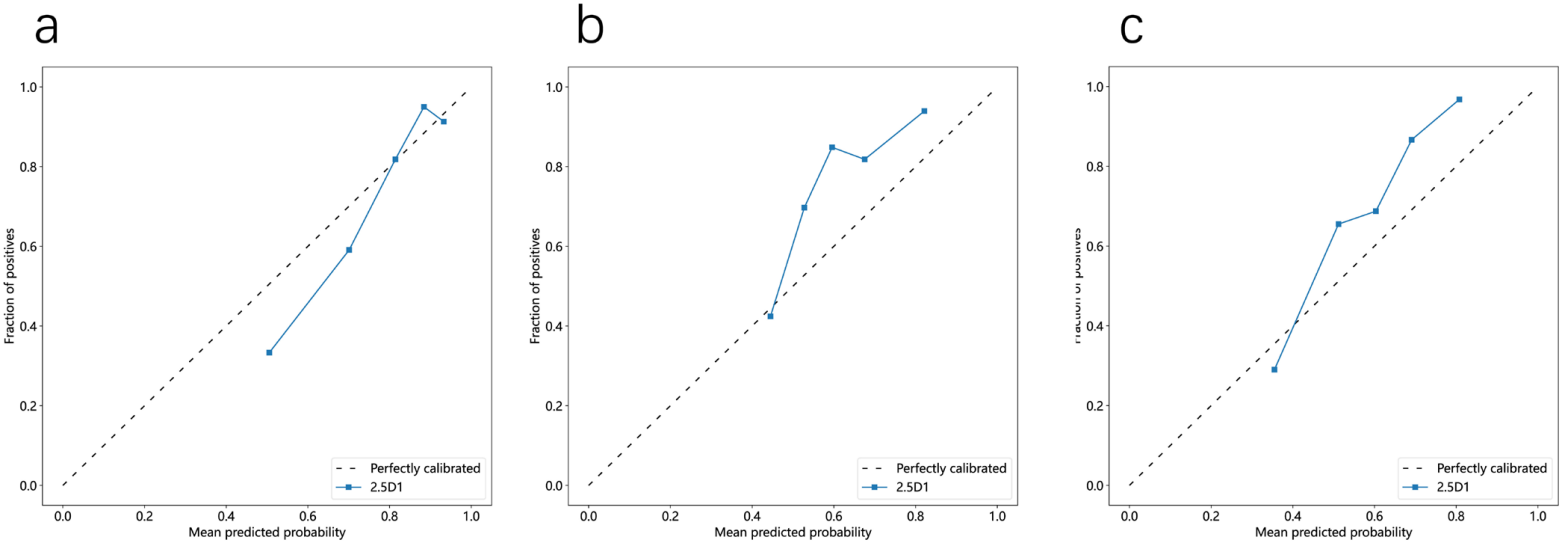
The calibration curves of the 2.5D1 (ROI original) deep learning models for PTR 4 mm in the training cohort (a), validation cohort 1 (b), and validation cohort 2 (c).

Similarly, we conducted a calibration analysis of the predictive model for the training cohort, validation cohort 1, and validation cohort 2 (Figure 9a–c). In the training cohort (Figure 9a), the calibration curve shows slight deviation in the lower prediction probability range, but it aligns closely with the ideal calibration line in the higher prediction probability range, indicating good calibration in the training cohort. In validation cohort 1 (Figure 9b), the calibration curve is similar to the ideal calibration line across most prediction probability ranges but shows some deviation in the higher prediction probability region, indicating reasonable calibration with minor deviations in validation cohort 1. The calibration curve for validation cohort 2 (Figure 9c) shows slight deviation from the ideal calibration line in the lower prediction probability range but aligns closely in the higher prediction probability range, indicating good calibration similar to validation cohort 1. In summary, calibration analysis indicates that the models exhibit good calibration across all datasets, with some deviations in specific prediction probability ranges but an overall trend consistent with the ideal calibration line, demonstrating reliable predictions across different datasets.

## 4 Discussion

This multicenter retrospective study of three distinct cohorts investigates the interplay between ROI dimensionality (2D/2.5D/3D), peritumoral expansion distances (0‐8 mm), and segmentation paradigms (ROI Only vs. Original) in deep transfer learning frameworks to distinguish luminal from non‐luminal breast cancer subtypes. The results indicate that the 2.5D1‐based deep learning model (ROI Original) achieved optimal performance with a 4 mm peritumoral expansion, with AUC of 0.766 (sensitivity 0.731, specificity 0.900, and accuracy 0.778) in the training cohort, 0.766 (sensitivity 0.707, specificity 0.690, and accuracy 0.703) in validation cohort 1, and 0.799 (sensitivity 0.689, specificity 0.766, and accuracy 0.712) in validation cohort 2.

Consistent with previous research findings [24, 25, 26, 27], this study reaffirms that the peritumoral region (tumor margin or boundary area) contains crucial information related to tumor growth and metastasis. The model performed better with a certain degree of peritumoral expansion, achieving optimal performance with a 4 mm expansion. Similarly, when constructing models that only retain the region of interest (ROI) while excluding background and normal tissue, the model's definition becomes more precise, but its performance does not necessarily improve. Therefore, when constructing models, it is necessary to balance precise definition with optimal performance, and adopt different approaches based on specific tasks.

Li et al. [28], Kim et al. [17], and Choi et al. [29] previously explored the advantages of 2.5D‐based deep learning models. 2.5D‐based deep learning models capture three‐dimensional information by integrating multiple consecutive two‐dimensional slices. Compared to single 2D‐based models, this method not only provides richer features but also integrates contextual information from individual slices, significantly enhancing the model's accuracy and robustness [30]. This approach is well suited for various medical imaging modalities as it captures and analyzes three‐dimensional information from 2D‐based slice sequences without requiring complete 3D data, making it particularly suitable for cases with incomplete data or challenging 3D reconstruction. In this study, despite the 3D‐based deep learning model incorporating more tumor region information and pretrained weights, its performance was still inferior to the 2.5D‐based model. This may be due to several factors: first, the 3D model handles higher‐dimensional data, which involves a larger number of parameters and greater training complexity, requiring more data and computational resources for adequate training even with pretrained weights. Additionally, the training of 3D‐based models demands more computational resources and time, which can result in insufficient training or suboptimal parameter optimization. Furthermore, 2.5D‐based models use 2D slices in each channel, potentially preserving more detail and resolution, whereas 3D models may lose some details when processing volumetric data. Moreover, the preprocessing and augmentation techniques for 3D data may not be as advanced as those for 2.5D data, affecting the training outcomes. In summary, 2.5D‐based deep learning models offer an effective approach for medical image analysis by balancing computational cost and information richness, capturing more three‐dimensional information than 2D‐based methods while being more efficient and adaptable than 3D‐based methods.

Among the different 2.5D‐based deep learning models, the model based on 2.5D1 performed the best, whereas the model based on 2.5D2 performed the worst. The reasons for this are likely as follows: the 2.5D1 method extracts three slices from the sagittal, coronal, and axial planes, capturing different features of the tumor in each plane and integrating information from various planes, thereby offering higher spatial information comprehensiveness and richness. In contrast, while the 2.5D2 method also adds some contextual information, its slices are primarily concentrated in a single direction, potentially neglecting important features in other directions, thereby limiting the model's understanding of the tumor's overall structure. Compared to the combination of slices from multiple directions, this approach offers lower correlation and effectiveness of information, resulting in relatively poorer model performance. Similarly, the 2.5D3 method improves model performance by capturing the most significant features from different perspectives. However, this method focuses on capturing the maximum features in each direction, which, although enhancing model performance in certain aspects, still falls short in terms of spatial information comprehensiveness. In summary, the method of using the three primary slices of the minimum bounding cube (2.5D1), as an approach approximating three‐dimensional reconstruction in medical imaging, enhances the model's predictive performance and clinical application value by providing rich spatial information and comprehensive structural features. This method captures features from more directions, allowing for a more accurate reflection of the tumor's overall structure, thus achieving the best performance among the deep learning models.

This study also has limitations. Although it included populations from different geographic regions, it is still a retrospective study, which may introduce selection bias and spectrum bias. Our study prioritized the peak enhancement phase (determined by time‐intensity curves) for DCE‐MRI analysis. The peak enhancement phase offers clinically interpretable and computationally efficient inputs for ROI analysis, particularly when compared with resource‐intensive full 4D modeling implementations. While our single‐phase strategy reduces operational complexity, it may obscure subtle temporal heterogeneity in contrast kinetics. This observation aligns with previous studies demonstrating enhanced diagnostic accuracy achieved through multiphase time‐series analysis [21, 31]. Future investigations will focus on developing hybrid architectures that integrate multiphase features to better characterize temporal dynamics while preserving clinical applicability. Additionally, because the included patients had a lower prevalence of HER2‐positive and triple‐negative breast cancer, this study only examined the performance differences of the prediction model in distinguishing luminal from nonluminal breast tumors. Future research should include a larger population to explore the adaptability of the prediction model in different tasks.

## 5 Conclusion

Under two segmentation scenarios (ROI only vs. ROI original), this study explored the effects of ROI dimensions (2D/2.5D/3D) and peritumoral expansion levels (0–8 mm) to optimize multidimensional deep learning models via transfer learning for distinguishing luminal from nonluminal breast cancers in DCE‐MRI‐based analysis. The 2.5D1 technique involves extracting slices from the sagittal, coronal, and axial planes, each emphasizing unique tumor characteristics. By integrating data from these various planes, the method enhances the comprehensiveness and richness of spatial information. Consequently, the 2.5D‐based deep learning model (ROI original, PRT 4 mm) demonstrated the best performance. This model can function as an effective diagnostic tool for distinguishing between luminal and nonluminal breast cancer tumors.

## Author Contributions

Z.H., Z.Q., and S.C. contributed equally to this work and are considered co‐first authors. Z.H.: conceptualization, methodology, writing – original draft. Z.Q.: data curation, formal analysis, visualization. S.C.: data curation, validation. Y.Z.: investigation, project administration. K.W.: investigation, project administration. Y.H.: data curation, validation. Y.Z.: supervision, writing – review and editing. J.B.: conceptualization, project administration. Q.Z.: resources, funding acquisition. J.B. and Y.Z. are co‐corresponding authors.

## Ethics Statement

This retrospective analysis has received approval from the ethics committees of the Fifth Affiliated Hospital of Sun Yat‐sen University (SYSU Hospital; approval number: K230‐1), Huizhou Central People's Hospital (HZ Hospital; approval number: ky112024007), and Linyi City People Hospital (LY Hospital; approval number: 202402‐H‐003). As this is a retrospective analysis, the requirement for patients' informed consent was waived.

## Conflicts of Interest

The authors declare no conflicts of interest.

## Supporting information


**Figure S1.** The ROI extraction methods of 2.5D1, 2.5D2, and 2.5D3.
**Figure S2.** 2D ROI original (a) and 2D ROI only (b).
**Figure S3.** 2.5D ROI original (a) and 2.5D ROI only (b).
**Figure S4.** 3D ROI original (a) and 3D ROI only (b).
**Figure S5.** Interaction between peritumoral expansions (0 mm–8 mm) and ROI dimensions (2D, 2.5D, and 3D) under the scenarios of ROI only.

## Data Availability

The data from the extended group are available upon request from the corresponding author. The data are not publicly available due to privacy or ethical restrictions.
