# Supplementary material for: Optimizing Deep Learning Models for Luminal and Nonluminal Breast Cancer Classification Using Multidimensional ROI in DCE‐MRI—A Multicenter Study
Source: Cancer Med. 2025 May 10;14(9):e70931. doi: 10.1002/cam4.70931 (PMC12065080; doi:10.1002/cam4.70931)
Supplement: Supplementary file 1 — Figure S1. The ROI extraction methods of 2.5D1, 2.5D2, and 2.5D3. Figure S2. 2D ROI original (a) and 2D ROI only (b). Figure S3. 2.5D ROI original (a) and 2.5D ROI only (b). Figure S4. 3D ROI original (a) and 3D ROI only (b). Figure S5. Interaction between peritumoral expansions (0 mm–8 mm) and ROI dimensions (2D, 2.5D, and 3D) under the scenarios of ROI only. [file CAM4-14-e70931-s001.docx]

**MRI data acquisition**

The Fifth Affiliated Hospital of Sun Yat-sen University

1、Equipment: 3.0T MRI Scanner (Model: GE SIGNA Pioneer), 8-channel phased-array body coil.

2、Dynamic Contrast-Enhanced Imaging (DCE):

（1）Sequence: T1-fl3d-tra-dynaVIEWS-1+7

（2）Scan Parameters: TR 4.37 ms, TE 1.68 ms, FA 10°, Matrix 384×384, Slice thickness 1.5 mm.

（3）Protocol: This sequence is a 3D spoiled gradient echo T1-weighted imaging applied for dynamic enhancement. It includes 1 baseline scan followed by 7 phases. After the first phase, a contrast agent (0.2 ml/kg Gd-DTPA) is injected intravenously within 2 minutes, followed by a 15 ml saline flush at a rate of 2.6 ml/s. Continuous non-interval scanning is performed across 7 phases, with each phase lasting 60 seconds.

Huizhou Central People's Hospital

1、Equipment: 3.0T MRI Scanner (Model: Siemens Magnetom Verio), 8-channel phased-array body coil.

2、Dynamic Contrast-Enhanced Imaging (DCE):

（1）Sequence: T1-fl3d-tra-dynaVIEWS-1+4

（2）Scan Parameters: TR 4.21 ms, TE 1.87 ms, FA 10°, Matrix 360×360, Slice thickness 1.0 mm.

（3）Protocol: This sequence is also a 3D spoiled gradient echo T1-weighted imaging applied for dynamic enhancement. It includes 1 baseline scan followed by 4 phases. After the first phase, a contrast agent (0.2 ml/kg Gd-DTPA) is injected intravenously within 8 seconds, followed by a 16 ml saline flush at a rate of 2.0 ml/s. Continuous non-interval scanning is performed across 4 phases, with each phase lasting 60 seconds.

Linyi City People Hospital

1、Equipment 1: United Imaging uMR780 3.0T MRI Scanner, 10-channel phased-array breast coil.

2、Dynamic Contrast-Enhanced Imaging (DCE):

（1）Sequence: T1-quick3d-tra-fs

（2）Scan Parameters: TR 4.72 ms, TE 2.14 ms, FA 10°, Matrix 400×320, Slice thickness 1.5 mm.

（3）Protocol: This sequence includes 1 baseline scan followed by 8 phases. After the first phase, a contrast agent (0.2 ml/kg Gd-DTPA) is injected intravenously within 15 seconds, followed by a 20 ml saline flush at a rate of 2.5 ml/s. Continuous non-interval scanning is performed across 8 phases, with each phase lasting 67 seconds.

1、Equipment 2: Philips Ingenia 3.0T MRI Scanner, ds Breast 7-channel breast coil.

2、Dynamic Contrast-Enhanced Imaging (DCE):

（1）Sequence: T1-FFE-3D-TRA-Dyn eTHRIVE-1+7, Dyn eTHRIVE+C TRA

（2）Scan Parameters: TR 3.60 ms, TE 1.85 ms, FA 11°, Slice thickness 2 mm, Matrix 260×341.

1、Equipment 3: GE Discovery MR750w 3.0T MRI Scanner (USA), 8-channel breast coil.

2、Dynamic Contrast-Enhanced Imaging (DCE):

（1）Sequence: Dynamic enhanced AX-VIBRANT (1+8 phases), with the first phase being the baseline scan followed by 8 dynamic enhancement phases.

（2）Protocol: After the first phase, a contrast agent (0.2 ml/kg, approximately 13 ml) is injected at a rate of 2 ml/s. Each phase lasts 1 minute and 1 second.

（3）Scan Parameters: TR 4.3 ms, TE 2.1 ms, FA 15°, Slice thickness 1.2 mm, Matrix 320×288.

**2.5D ROI extraction Methods**

2.5D1: Minimum Bounding Cube (MBC) Main Slice Combination

1、Identify the MBC: Determine the minimum bounding cube that encloses the region of interest (ROI) within the 3D image data.

2、Extract Main Slices: From the MBC, extract the sagittal, coronal, and axial slices that pass through the center of the ROI.

3、Form Multi-Channel Image: These three main slices are combined into a multi-channel image, with each slice representing a different channel (e.g., RGB).

4、Resize: Before combining, resize each slice to 224×224 pixels.

This method integrates major slices from different planes, providing comprehensive spatial information.

2.5D2: Central and Adjacent Slices Combination

1、Select Central Slice: Identify the central slice (nth slice) of the ROI based on the largest cross-sectional area.

2、Select Adjacent Slices: Choose the slice two slices above (n-2) and two slices below (n+2) the central slice.

3、Form Multi-Channel Image: Combine these three slices into a multi-channel image, with each slice as a different channel.

4、Resize: Resize each slice to 224×224 pixels before combining.

This approach combines the central slice with its immediate neighbors, providing additional context.

2.5D3: Multi-View Maximum Cross-Section Combination

1、Identify Maximum Cross-Sections: From the sagittal, coronal, and axial view slice sequences, find the slice with the largest ROI area in each view.

2、Extract Maximum Slices: Extract these maximum cross-section slices from each view.

3、Form Multi-Channel Image: Combine these three slices into a multi-channel image, each representing a different channel.

4、Resize: Resize each slice to 224×224 pixels before combining.

This method captures the maximum ROI areas from multiple views, offering diverse spatial information.

The detailed steps and processes of these three methods are illustrated in appendix Fig. 1.By employing these methods, each utilizing different aspects of 2.5D imaging, researchers can leverage a combination of comprehensive spatial information, contextual details, and multi-directional perspectives to enhance the performance of deep learning models in medical image analysis.

Appendix Fig. 1 The ROI Extraction Methods of 2.5D1、2.5D2 and 2.5D3

**Definition of ROI Only**

2D ROI Only Processing

1、Select Maximum Cross-Section: Choose the image slice with the largest cross-section of the tumor.

2、Adjust Minimum Bounding Rectangle (MBR): Adjust the MBR to encompass only the tumor tissue, excluding surrounding normal tissue and background.

3、Retain Tumor Region: Keep only the tumor region within the MBR for further analysis.

This method enhances the model's precision in identifying and analyzing tumor boundaries by focusing exclusively on the tumor region in the 2D plane. Appendix Fig. 2 shows the differences between 2D ROI Original and 2D ROI Only.

Appendix Fig. 2 2D ROI Original (a) and 2D ROI Only (b)

2.5D ROI Only Processing

1、2D ROI Only Slices: Use the slices obtained from the 2D ROI Only method.

2、Combine Slices: Integrate the 2D ROI Only slices into a multi-channel image, similar to the 2.5D processing methods.

3、Retain Tumor Regions: Retain only the ROI regions from the 2D slices, excluding background and normal tissue.

By combining the advantages of 2D and 3D methods, 2.5D ROI Only processing leverages detailed information from multiple layers of 2D slices to enhance the overall recognition capability of the model.Appendix Fig. 3 shows the differences between 2.5D ROI Original and 2.5D ROI Only.

These ROI Only methods ensure precise segmentation and focus on the tumor region, improving the accuracy and reliability of radiomics models. They eliminate irrelevant data from normal tissue and background, which can interfere with model performance.

Appendix Fig.3 2.5D ROI Original (a) and 2.5D ROI Only (b)

3D ROI Only Processing

1、Identify Minimum Bounding Cube (MBC): Determine the MBC that encloses the ROI within the 3D image data.

2、Adjust MBC: Modify the MBC to retain only the tumor region within it, excluding normal tissue and background.

3、Retain Tumor Region: Keep only the tumor region within the MBC for further analysis.

This step ensures the tumor region is precisely defined in the 3D space, enhancing the accuracy of the 3D analysis.Appendix Fig. 4 shows the differences between 3D ROI Original and 3D ROI Only.

Appendix Fig. 4 3D ROI Original (a) and 3D ROI Only (b)

This schematic diagram illustrates the interaction between Peritumoral Expansions (0mm–8mm) and **R**OI Dimensions (2D, 2.5D, 3D) under the scenarios of ROI only (Appendix Fig. 5). This schematic diagram's core mechanism remains applicable under ROI original scenarios.


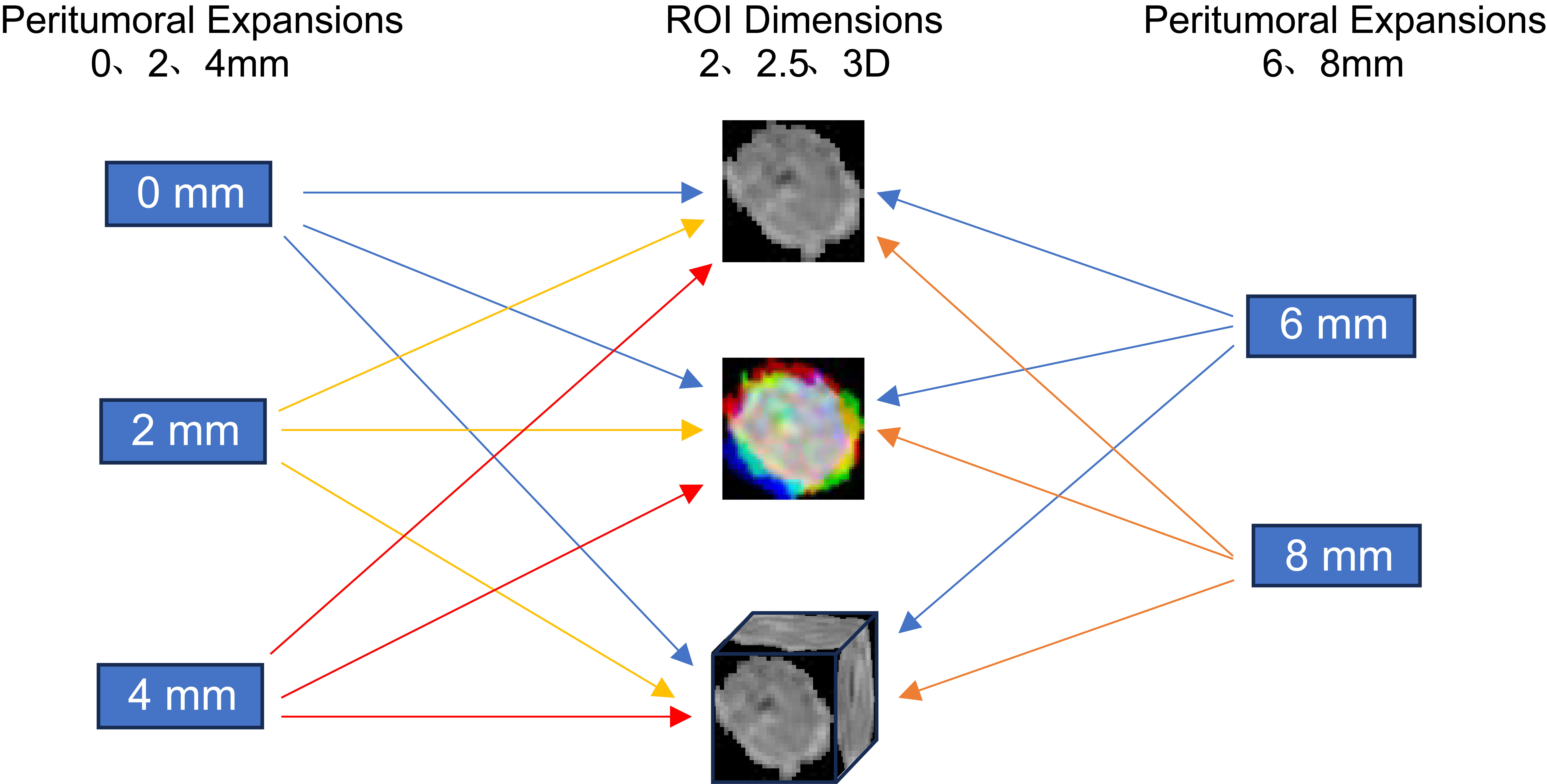


Appendix Fig. 5 Interaction between Peritumoral Expansions (0mm–8mm) and ROI Dimensions (2D, 2.5D, 3D) under the scenarios of ROI only
